# Inequality in total fertility rates and the proximate determinants of fertility in 21 sub-Saharan African countries

**DOI:** 10.1371/journal.pone.0203344

**Published:** 2018-09-18

**Authors:** Jocelyn E. Finlay, Iván Mejía-Guevara, Yoko Akachi

**Affiliations:** 1 Department of Global Health and Population, Harvard TH Chan School of Public Health, Boston, Massachusetts, United States of America; 2 Department of Biology, Stanford University, Stanford, California, United States of America; 3 Stanford Medicine Center for Population Health Sciences, Palo Alto, California, United States of America; 4 Independent Consultant, Helsinki, Finland; The University of Warwick, UNITED KINGDOM

## Abstract

In this paper, we examine the inequality in the dynamics of the total fertility rate *within* 21 sub-Saharan African countries by wealth quintiles. We also examine the associated inequality within each country in the proximate determinants of fertility–marriage, contraception, and breastfeeding. Applying Bongaarts’ proximate determinants of fertility framework, for 14/21 countries we analyze, we find that those in the richest wealth quintiles have had a more rapid decline in fertility rates than those in the poorest wealth quintiles. The rapid decline for those in the richest wealth quintiles is attributable to delayed marriage and modest increases in contraceptive use. Although the poorest lag in fertility decline, postpartum abstinence and breastfeeding are the most important factors for them for fertility regulation. Further encouraging maternal health programs that focus on natural methods of fertility regulation will work in favor of the poorest in sub-Saharan Africa in moving through the demographic transition.

## Introduction

Globally, countries have seen a decline in their national-level total fertility rates over the past 100 years, although this decline has begun in different years and evolved at different rates. The variation across countries in the rate and timing of fertility decline may be a reflection of variations *within* a country’s fertility rate dynamics. Examination of the fertility rates at the sub-national level can inform us of inequalities that may hinder the decline in the national-level fertility rates. For example, the richest declining rapidly in their fertility rates, but the poorest remaining stagnant or increasing in their fertility rates. At the aggregate level, reflected in the national-level fertility rates, it would appear that fertility decline is slow for everyone. Rather, there is great variation across the wealth spectrum within a country in the rate and timing of fertility decline. Examination at the sub-national level reveals these within-country inequalities in the total fertility rate dynamics. Furthermore, inequalities in the total fertility rate reflect inequalities in the proximate determinants of fertility: child marriage, access to contraception and safe abortion, and postpartum breastfeeding or abstinence, can vary within countries across wealth quintiles. Examination of these proximate determinants of fertility can reveal underling drivers of inequality in the total fertility rates across subgroups of the national population.

The total fertility rate as a calculation is the number of children born per woman if she were to age through her childbearing years and have children according to the current schedule of age-specific fertility rates. It is not the number of children a woman gives birth to, but a hypothetical projection of what she would give birth to if her own age-specific fertility patterns across her life course matched the population average age-specific patterns in the present.

An inequality in the total fertility rate refers to an observable and measurable difference between or among individuals, subgroups of the population, or groups occupying unequal positions in society [1].

A decline in the total fertility rate to the replacement rate of 2.1 children per women has been shown to have a positive impact on a nation’s economic prosperity (the demographic dividend [2–4]). The consideration of the total fertility rate is usually reported at the national level, and national-level declines in fertility then translate to national-level economic benefits. While the consideration of sub-national inequalities in the total fertility rate and the subsequent impact on economic inequality has been little explored [5, 6], that line of research examines the *consequences* of inequality in the total fertility rates. In this paper, however, we examine the existence of inequality in the total fertility rate within countries, and the proximate *determinants* of this inequality in the total fertility rates. We leave the examination of the consequences of inequality in fertility rate on inequality in economic development for another paper.

Our examination of the inequality in the total fertility rate at the sub-national level, and investigation of the drivers of this inequality in the total fertility rates, enables us to observe the underlying pathways of how inequality in age of marriage, contraceptive use, and breastfeeding affects inequality in the total fertility rate.

At the sub-national level, the examination of inequality in the total fertility rate has been observed within countries by education levels [7]. Eloundou and his co-authors use education as one of the social determinants of fertility and find that the most educated women within the country drive an observed fall in the total fertility rate. However, this research does not extend the descriptive analysis to the observation of the role the underlying proximate determinants of fertility play in shaping the observed inequality in the fertility rate.

As a result, in this paper we conduct a descriptive data analysis on individual-level data to illustrate inequality in changes in the total fertility rates within subgroups (wealth quintiles) of a country’s population. We use Bongaarts’ [8] model of the proximate determinants of fertility to examine these questions of inequality in fertility rates and inequality in the proximate determinants of fertility–delayed marriage, contraceptive use, abortion, and postpartum infecundability (postpartum abstinence and breastfeeding). Note that Bongaarts’ model enables a way of looking at the data in a descriptive representation, but does not establish a casual relationship.

Bongaarts’ [8] model of the proximate determinants of fertility is an updated version of Bongaarts’ [9] and Stover’s [10] models. Bongaarts’ 1982 model, which is based on the one outlined by Davis and Blake [11], showed that empirically a set of intermediate variables can be reduced to a concise list of four factors: proportion of women married, postpartum infecundability, contraception, and induced abortion. That is, fertility is lower than the biological maximum because of delayed marriage, the use of contraception or abortion, and postpartum infecundability due to breastfeeding or abstinence. Across time, if the total fecundity rate remains unchanged then changes in the total fertility rate can be attributed to changes in these four key variables. In other words, extended periods of postpartum infecundability, abortion, contraceptive use, and delayed marriage all play a part in reducing the observed total fertility rate from the biological maximum.

While Bongaarts’ model has received its share of citicism [12, 13], we found that by applying this model to the data we were able to include each of the proximate determinants with a theoretical justification rather than simply generating an ad hoc list to use in a regression analysis. Because the model provides a comprehensive way to quantify the role of the proximate determinants of fertility in shaping the total fertility rate, we can calculate the relative contribution of each of the proximate determinants in shaping fertility levels and trends.

The social determinants of fertility are education, wealth, and urban/rural living. In this paper, we consider the role of wealth as a moderator of the relationship between the proximate determinants of fertility and the total fertlity rate. We hypothesize that we will observe differences in the roles of the proximate determinants of fertility across wealth quintiles within a country, which will illustrate the underlying reason for differences in the total fertility rate across wealth quintiles within a country.

In this paper, we aim to show: 1) the total fertility rate by wealth quintiles over time, 2) inequality in the total fertility rates over time, 3) the proximate determinants of fertility by wealth quintile over time; and 4) inequality in the proximate determinants of fertility over time.

This decomposition of the data enables us to see which countries are experiencing inequality in fertility decline and what is driving this inequality. The decomposition of the proximate determinates enables us to observe a side-by-side comparison where policy can be most effective. For example, we can see how policies ending child marriage, increasing contraceptive use, or promoting natural methods of fertility regulation could be promoted, and which groups within a country would benefit most from such policies.

In this paper we focus on the sub-Saharan African continent, as their demographic transition is beginning, but is unfolding slowly [14] and its determinants are worth investigating independently from other regions [15] as even across (and within) countries, marriage, contraceptive use and breastfeeding can differ.

It is worth noting that a large-scale secondary source dataset such as the Demographic and Health Surveys, which interviews across different time points or different countries, have varied in quality. Furthermore, the methods for calculating the total fertility rate published by the national statistical office or the United Nations, or total fertility rates by wealth quintile published in DHS Final Reports may differ from our estimation methods. Moreover, our sample may differ from the sample used in the DHS Final Reports, as we only include women who respond to questions regarding wealth, birth history, sexual union, contraceptive use, postpartum abstinence *and* breastfeeding. Thus, there may be differences in our reported numbers to the fertility rates reported by others, as our sample is restricted to women who respond to all these questions.

## Methods

### Data

In this paper we used data from the Demographic and Health Surveys (DHS) [16, 17], which are collected from over 90 low- and middle-income countries, including 44 sub-Saharan African (SSA) countries. The data were nationally representative. From a household roster, women aged 15–49 were identified for an in-depth interview, and there was detailed information on fertility, contraceptive use, and reproductive health of each woman recorded. Since 1987 there have been six phases of the DHS. Many countries had multiple surveys (note it is a repeated cross section, not a panel), and in SSA there were 23 countries with three or more DHS standard surveys. We chose countries with three or more surveys, so that we could see trends over time rather than just a line between two points. In an earlier working paper [18], we presented the trends across time highlighting the usefulness of having more than two points in time. Wealth index information was collected from Phase II, and 21 SSA countries had three or more surveys and included wealth information. All women aged 15–49 years old were included in this analysis if the information was available for each of the variables.

The 21 countries included were Benin, Burkina Faso, Cameroon, Cote d’Ivoire, Ethiopia, Ghana, Guinea, Kenya, Madagascar, Malawi, Mali, Mozambique, Namibia, Niger, Nigeria, Rwanda, Senegal, Tanzania, Uganda, Zambia, and Zimbabwe. See Table 1.

**Table 1:** Countries and surveys in the sample.

			Survey years				
	N	Number of surveys	1990–1994	1995–1999	2000–2004	2005–2009	2010–2014
Benin	38,199	4		1996	2001	2006	2011
Burkina Faso	35,405	4	1992	1998	2003		2010
Cameroon	28,930	4	1991	1998	2004		2011
Cote d'Ivoire	17,563	3	1994	1998			2011
Ethiopia	30,443	3			2000	2005	2010
Ghana	18,790	4	1993	1998	2003	2008	
Guinea	20,359	3		1999		2005	2012
Kenya	29,082	4	1993	1998	2003	2008	
Madagascar	30,229	3	1997		2003	2008	
Malawi	41,605	4	1992		2000, 2004		2010
Mali	44,687	4		1995	2001	2006	2012
Mozambique	29,503	3		1997	2003		2011
Namibia	23,626	4	1992		2000	2006	2013
Niger	29,087	3		1998		2006	2012
Nigeria	76,737	4	1990		2003	2008	2013
Rwanda	24,307	4	1992		2000	2005	2010
Senegal	49,189	5	1997			2005	2010, 2012, 2014
Tanzania	32,920	4	1996	1999	2004	2009	
Uganda	27,951	4		1995	2000	2006	2011
Zambia	35,043	4		1996	2001	2007	2013
Zimbabwe	30,853	4	1994	1999		2005	2010
TOTAL	694,508						

Source: Authors’ calculation based on DHS data.

The outcome of interest is the total fertility rate. This is estimated using two methods. The first uses the Rodriguez’s Stata code (http://data.princeton.edu/eco572/asfr.html) applying birth histories from the DHS to calculate the age-specific fertility rate in the three-year period before the survey. The second method for calculating the total fertility rate is an application of the Bongaarts’ proximate determinants of fertility model [8]. Such that, fertility rates are a multiplicative function of exposure to sexual activity, contraceptive use, abortion, postpartum infecundability, and an unexplained residual. This residual refers to the factors that directly affect the total fertility rate but are not observable in the current data set or modelled.

We examine the total fertility rate and the proximate determinants of fertility across the 21 sub-Saharan African countries and by wealth quintile within each country, over multiple surveys (time points). The wealth quintiles calculated by the DHS [19] are a compilation of each household’s assets, and the first principal component is used as the information for the wealth index. Households with more assets had higher scores. The scores were assigned by household, then ranked, and then divided into equal population quintiles: five groups with the same number of households in each (http://www.dhsprogram.com/topics/wealth-index/Index.cfm).

### Summary of Bongaarts’ model

In this study, we followed Bongaarts’ [8] model of the proximate determinants of fertility. The Bongaarts’ framework provides an understanding of the four main proximate determinants of fertility in shaping levels and trends in the total fertility. In Bongaarts’ model, the total fertility rate can be estimated to be a function of the biological maximum total fertility (TF). Then, delayed exposure to sexual activity (Cm), contraceptive use (Cc), abortion (Ca), postpartum infecundability (Ci), and the residual reduce the fertility rate from the biological maximum to the observed rate.

Delaying marriage or delayed initiation of sexual activity will reduce the years that a woman of reproductive age is exposed to the risk of pregnancy. Contraceptive use decreases the risk of pregnancy. Abortion reduces the number of live births. Postpartum infecundability is a combination of postpartum abstinence and postpartum amenorrhea. The latter is a result of breastfeeding following a birth.

The proximate determinant variables point directly to policy instruments that are relevant in the maternal health space: child marriage, low contraceptive use, high rates of unsafe abortions, and the need for support in the post-partum period. All are issues that plague women, particularly in low-resource settings. Bongaarts’ papers [8, 9, 20], and our own working paper [18], provide details of the calculations of the proximate determinants of fertility.

Each of the proximate determinants work to reduce fertility from the biological maximum, which we set at 15.3 following Bongaarts’ earlier paper [20]. If the age of marriage or sexual debut is delayed, then this contributes toward an observed decline in the fertility rate. If contraceptive use (or effectiveness) rates increase, this too contributes to an observed decline in the total fertility rate. Following a birth, the period of postpartum amenorrhea may be extended due to a longer period of intensive breastfeeding or postpartum abstinence, and will act to reduce the total fertility rate. The model also accounts for abortion rates, but we did not have sufficient data at the country or sub-national level and instead applied region-specific aggregate rates estimated by Sedgh, Singh et al. [21]. Abortion rates are in part a function of postpartum infecundability, as a woman may have a period of abstinence following an abortion and this is accounted for in the Bongaats’ estimation of the contribution of abortion rates in determining fertility rates. In the estimations in this study, a period of postpartum infecundability does vary by wealth quintile at the sub-national level, thus the descriptive data is not constant within regions even though the abortion rates are constant at the regional level.

We examine trends in the total fertility rate, and the proximate determinants, by wealth quintile, following the approach of Majumder and Ram [22] who focused on the South Asian region, and early work by Finlay et al [18].

## Results

### Total fertility rate by wealth quintile, over time

In this paper, we calculate the total fertility rate by wealth quintile using Rodriguez’s code for age-specific fertility rates. Table 2 (poorest–richest columns) shows that the total fertility rate of the poorest is higher than the total fertility rate of the richest for all 21 countries. In the earliest surveys, the poorest have total fertility rates as high as 9.4 children per woman in Rwanda in 1992, and the lowest rate is 6.4 children per woman in Cameroon and Guinea. By the most recent survey, the total fertility rate for the poorest ranged from 5.7 in Zimbabwe 2010 to 8.7 in both Uganda 2011 and Niger 2012.

**Table 2:** Levels and changes in the total fertility rates by richest and poorest, earliest and latest surveys.

	TFR Rodriguez								
	Earliest Year			Latest Year					
	Poorest (1)	Richest (2)	Poorest–Richest (3)	Poorest (4)	Richest (5)	Poorest–Richest (6)	Latest Poorest—Earliest Poorest (7)	Latest Richest—Earliest Richest (8)	Latest (Poorest—Richest)—Earliest (Poorest—Richest) (9)
Benin	8	4.7	3.3	6.9	4.7	2.2	-1.1	0	-1.1
Burkina Faso	8	5.5	2.5	7.7	4.3	3.4	-0.3	-1.2	0.9
Cameroon	6.4	5.1	1.4	7.9	3.7	4.1	1.4	-1.3	2.8
Cote d'Ivoire	7.2	4.7	2.5	7.1	3.8	3.3	-0.2	-1	0.8
Ethiopia	7.8	4.5	3.4	7.7	3.9	3.8	-0.1	-0.6	0.5
Ghana	7.5	4	3.5	7.4	3.2	4.2	-0.2	-0.9	0.7
Guinea	6.4	4.7	1.7	6.9	4.1	2.8	0.5	-0.6	1.1
Kenya	8.2	4.4	3.8	8.2	4	4.2	0	-0.4	0.4
Madagascar	9.1	4.1	5	7.4	3.3	4.1	-1.7	-0.8	-0.9
Malawi	7.9	6.5	1.4	7.9	4.7	3.2	0	-1.7	1.8
Mali	7.8	6.2	1.6	7.4	5.5	2	-0.4	-0.7	0.3
Mozambique	6.6	4.9	1.7	8.1	3.8	4.3	1.5	-1	2.5
Namibia	7.2	4.1	3.2	5.9	2.7	3.2	-1.3	-1.3	0
Niger	9.1	7	2.2	8.7	6.9	1.8	-0.4	-0.1	-0.3
Nigeria	7	5.8	1.2	7.8	4.6	3.1	0.7	-1.2	1.9
Rwanda	9.4	6.8	2.6	7.5	5.5	1.9	-1.9	-1.3	-0.7
Senegal	8.2	5.4	2.8	8.2	4.8	3.4	-0.1	-0.7	0.6
Tanzania	8.1	4.5	3.6	7.6	4.6	3	-0.5	0.1	-0.6
Uganda	7.6	5.9	1.7	8.7	5.1	3.6	1.1	-0.8	1.9
Zambia	7.7	5.8	1.9	8.4	4.4	4	0.7	-1.3	2
Zimbabwe	7.2	4	3.2	5.7	3.4	2.3	-1.4	-0.6	-0.9

Source: Authors’ calculation based on DHS data.

Note: (1) Levels of TFR for the poorest in earliest survey, (2) Levels of TFR for the richest in earliest survey, (3) Gap between rich and poor in earliest survey, (4) Levels of TFR for the poorest in latest survey, (5) Levels of TFR for the richest in latest survey, (6) Gap between rich and poor in latest survey, (7) Change in poorest TFR over time, (8) Change in richest TFR over time, (9) Change in the gap between rich and poor over time.

Over time, for the poorest quintile (Table 2, Latest Poorest–Earliest Poorest), 13/21 countries saw the total fertility rate *decline*. This decline ranged from a drop of 1.9 children per woman for the poorest in Rwanda between 1992 and 2010, to a 0.1 drop in the number of children per woman in Ethiopia (7.8 in 2000 to 7.7 in 2010) and Senegal (8.2 in 1997 to 8.2 in 2014, with rounding). In the case of Rwanda, Rutayisire et al [23] explore the changing decomposition of this fertility decline over time. They found contraceptive use a driving force in later years, but delayed marriage playing a role in earlier years. Two countries, Kenya and Malawi, saw *no change* in the total fertility rate of the poorest wealth quintile over time. For six countries, Cameroon, Guinea, Mozambique, Nigeria, Uganda, and Zambia, the total fertility rate of the poorest *increased* from the earliest to the latest survey. The poorest in Mozambique had the largest absolute increase in the total fertility rate, from 6.6 children per woman in 1997 to 8.1 children per woman in 2011.

Over time, for the richest quintile (Latest Richest–Latest Poorest), 19/21 countries saw a decrease in their total fertility rate. Benin saw no change in the total fertility rate of the richest over time. Tanzania saw an increase of 0.1 children per woman for the richest quintile between 1996 and 2009.

### Inequality in total fertility rates increasing or decreasing over time

Table 2 also shows the difference in the total fertility rate (Rodriguez estimate) of the poorest wealth quintile minus the richest wealth quintile, in the earliest and latest survey years. We took the earliest and latest survey for each of the countries in our sample and looked at the change in the *gap* in total fertility rate of the poorest minus the total fertility rate of the richest. We found that for 14 of the 21 countries, the gap between the poorest and richest total fertility rate increased. That is, inequality in the total fertility rate increased for these 14 countries: Burkina Faso, Cameroon, Cote d’Ivoire, Ethiopia, Ghana, Guinea, Kenya, Malawi, Mali, Mozambique, Nigeria, Senegal, Uganda, and Zambia.

The gap between poorest and richest was constant in Namibia. For the remaining six countries in the sample, the gap between poorest and richest declined (Benin, Madagascar, Niger, Rwanda, Tanzania, and Zimbabwe).

Of the 14 countries that saw an increase in inequality in fertility decline, for six countries this was due to more rapid declines for the richest compared to the slower declines of the poorest quintiles over time (Burkina Faso, Cote d’Ivoire, Ethiopia, Ghana, Mali, and Senegal). For another 6/14 countries that saw an increase in fertility inequality, this rise in inequality was due to the poorest experiencing an increase in the total fertility rate while the richest experienced a decline in the total fertility rate over time (Cameroon, Guinea, Mozambique, Nigeria, Uganda, and Zambia). For Kenya and Malawi, there was an increase in inequality of the fertility rate due to a decline in the total fertility rate over time for the richest, but no change in the total fertility rate over time for the poorest.

For Namibia, the total fertility rate decreased by the same amount for the richest and poorest, (falling by 1.3 points) and so the gap between rich and poor remained the same over time. The poorest had a total fertility rate of 7.2 in the earliest survey, and the richest had a total fertility rate of 4.1. By the time of the most recent Namibian survey, the total fertility rate was 5.9 for the poorest (down 1.3) and 2.7 (down 1.3) for the richest.

For the six countries that saw a closing of the gap between the richest and poorest total fertility rates over time, four countries did so due to the decline in fertility of the poorest being greater than the decline in fertility of the richest (Madagascar, Niger, Rwanda, and Zimbabwe). For Benin, the poorest saw a decline in the total fertility rate over time, while the richest saw no change. For Tanzania, the poorest saw a decline in the total fertility rate over time, while the richest saw an increase in their total fertility rate over time.

### Proximate determinants of fertility by wealth quintile, over time

Thus far, we have examined how the gap between rich and poor in the total fertility rate has widened (increasing inequality in fertility decline) or narrowed (decreasing inequality in fertility decline) over time. Furthermore, we examined whether this widening inequality is due to the poor lagging behind or the rich accelerating ahead.

We now turn to examine how the proximate determinants of fertility–marriage, contraception, and postpartum abstinence/amenorrhea, play a role in shaping the widening or narrowing fertility inequality.

Tables 3 and 4 show how each of the proximate determinants of fertility reduces fertility from the biological maximum (15.3) to the observed total fertility rate by wealth quintiles, as estimated by the Rodriguez code of calculating the age-specific fertility rate using the DHS birth histories.

**Table 3:** Proximate determinants of fertility by wealth quintile, earliest survey.

	Poorest						Richest						Poorest–Richest					
		R	M	C	A	I	TFR	R	M	C	A	I	TFR	R	M	C	A	I
Burkina Faso	8.0	**0.1**	0.5	0.1	0.5	6.1	5.5	**0.8**	1.0	1.3	0.8	6.0	2.5	**-0.7**	-0.5	-1.2	-0.3	0.2
Cote d'Ivoire	7.2	**1.5**	0.6	0.1	0.7	5.1	4.7	**1.8**	1.1	1.9	1.1	4.5	2.5	**-0.3**	-0.5	-1.8	-0.4	0.5
Ethiopia	7.8	**-1.9**	2.6	0.1	0.5	6.1	4.5	**-0.5**	3.0	1.7	1.0	5.7	3.4	**-1.4**	-0.4	-1.6	-0.5	0.5
Ghana	7.5	**0.3**	1.2	0.5	0.7	5.1	4.0	**2.0**	1.7	2.0	1.4	4.2	3.5	**-1.7**	-0.5	-1.5	-0.7	0.9
Mali	7.8	**1.8**	0.6	0.1	0.7	4.4	6.2	**1.2**	1.2	1.8	1.0	4.1	1.6	**0.6**	-0.6	-1.7	-0.2	0.3
Senegal	8.2	**0.0**	0.9	0.1	0.6	5.6	5.4	**-0.8**	3.6	1.6	1.0	4.5	2.8	**0.7**	-2.7	-1.5	-0.4	1.1
**Group average**	**7.8**	**0.3**	**1.1**	**0.2**	**0.6**	**5.4**	**5.1**	**0.7**	**1.9**	**1.7**	**1.0**	**4.8**	**2.7**	**-0.4**	**-0.9**	**-1.6**	**-0.4**	**0.6**
Kenya	8.2	**-0.6**	1.4	0.7	0.7	5.0	4.4	**0.2**	1.8	3.5	1.4	4.0	3.8	**-0.8**	-0.4	-2.8	-0.7	1.0
Malawi	7.9	**-0.4**	2.2	0.5	0.8	4.3	6.5	**0.1**	1.8	1.8	1.0	4.1	1.4	**-0.5**	0.4	-1.3	-0.2	0.2
**Group average**	**8.0**	**-0.5**	**1.8**	**0.6**	**0.8**	**4.6**	**5.4**	**0.1**	**1.8**	**2.7**	**1.2**	**4.1**	**2.6**	**-0.6**	**0.0**	**-2.1**	**-0.5**	**0.6**
Cameroon	6.4	**2.0**	0.6	0.1	0.7	5.5	5.1	**2.0**	0.7	2.0	1.0	4.6	1.4	**0.0**	0.0	-1.9	-0.3	0.9
Guinea	6.4	**1.4**	0.5	0.1	0.4	6.5	4.7	**1.4**	1.2	1.0	0.6	6.4	1.7	**0.0**	-0.7	-1.0	-0.2	0.2
Mozambique	6.6	**2.3**	1.0	0.1	0.9	4.5	4.9	**1.9**	1.2	1.5	1.2	4.6	1.7	**0.4**	-0.2	-1.4	-0.3	-0.2
Nigeria	7.0	**1.2**	0.7	0.0	0.6	5.7	5.8	**0.9**	1.1	1.3	0.8	5.3	1.2	**0.3**	-0.4	-1.3	-0.2	0.3
Uganda	7.6	**1.2**	0.7	0.2	0.8	4.8	5.9	**0.7**	1.3	2.3	1.1	4.0	1.7	**0.5**	-0.6	-2.0	-0.3	0.8
Zambia	7.7	**0.0**	1.2	0.5	0.7	5.2	5.8	**-1.0**	2.4	2.6	1.1	4.4	1.9	**1.0**	-1.2	-2.1	-0.4	0.8
**Group average**	**7.0**	**1.3**	**0.8**	**0.2**	**0.7**	**5.4**	**5.3**	**1.0**	**1.3**	**1.8**	**1.0**	**4.9**	**1.6**	**0.4**	**-0.5**	**-1.6**	**-0.3**	**0.5**
Namibia	7.2	**0.6**	2.0	0.4	0.4	4.6	4.1	**0.4**	1.2	4.6	0.7	4.4	3.2	**0.2**	0.9	-4.2	-0.3	0.3
**Group average**	**7.2**	**0.6**	**2.0**	**0.4**	**0.4**	**4.6**	**4.1**	**0.4**	**1.2**	**4.6**	**0.7**	**4.4**	**3.2**	**0.2**	**0.9**	**-4.2**	**-0.3**	**0.3**
Madagascar	9.1	**-0.4**	1.1	0.2	0.7	4.5	4.1	**1.2**	2.2	2.7	1.7	3.4	5.0	**-1.6**	-1.1	-2.5	-1.0	1.1
Niger	9.1	**-0.2**	0.8	0.0	0.6	4.9	7.0	**-0.3**	2.1	1.5	0.8	4.3	2.2	**0.2**	-1.3	-1.4	-0.3	0.7
Rwanda	9.4	**-3.2**	2.7	0.7	0.6	5.0	6.8	**-2.2**	3.7	1.5	0.8	4.6	2.6	**-0.9**	-0.9	-0.8	-0.2	0.4
Zimbabwe	7.2	**-0.3**	1.6	1.7	0.9	4.3	4.0	**-0.6**	2.2	4.1	1.5	4.1	3.2	**0.3**	-0.6	-2.4	-0.7	0.2
**Group average**	**8.7**	**-1.0**	**1.6**	**0.7**	**0.7**	**4.7**	**5.5**	**-0.5**	**2.6**	**2.4**	**1.2**	**4.1**	**3.2**	**-0.5**	**-1.0**	**-1.8**	**-0.5**	**0.6**
Benin	8.0	**0.7**	0.5	0.2	0.6	5.3	4.7	**1.9**	1.4	1.7	1.2	4.4	3.3	**-1.3**	-0.8	-1.4	-0.6	0.8
**Group average**	**8.0**	**0.7**	**0.5**	**0.2**	**0.6**	**5.3**	**4.7**	**1.9**	**1.4**	**1.7**	**1.2**	**4.4**	**3.3**	**-1.3**	**-0.8**	**-1.4**	**-0.6**	**0.8**
Tanzania	8.1	**-0.2**	0.9	0.4	0.6	5.5	4.5	**1.2**	1.0	2.2	1.2	5.1	3.6	**-1.5**	-0.2	-1.9	-0.5	0.4
Group average	8.1	**-0.2**	0.9	0.4	0.6	5.5	4.5	**1.2**	1.0	2.2	1.2	5.1	3.6	**-1.5**	-0.2	-1.9	-0.5	0.4
Total average	7.7	0.3	1.2	0.3	0.7	5.1	5.2	0.6	1.8	2.1	1.1	4.6	2.6	-0.3	-0.6	-1.8	-0.4	0.5

Note: TFR = total fertility rate, R = residual, M = Cm marriage and exposure index, C = Cc contraceptive use index, A = Ca abortion index, I = Ci postpartum infecundability index

**Table 4:** Proximate determinants of fertility by wealth quintile, latest survey.

	Poorest						Richest						Poorest—Richest					
	TFR	R	M	C	A	I	TFR	R	M	C	A	I	TFR	R	M	C	A	I
Burkina Faso	7.7	**0.5**	1.1	0.4	0.5	5.1	4.3	**1.1**	1.5	2.9	1.0	4.4	3.4	**-0.6**	-0.4	-2.5	-0.5	0.7
Cote d'Ivoire	7.1	**1.7**	0.8	0.5	0.6	4.7	3.8	**2.4**	1.7	2.1	1.1	4.2	3.3	**-0.7**	-0.9	-1.7	-0.6	0.5
Ethiopia	7.7	**-1.0**	2.0	0.7	0.6	5.3	3.9	**-0.8**	2.6	3.9	1.4	4.3	3.8	**-0.2**	-0.6	-3.2	-0.8	1.0
Ghana	7.4	**-0.3**	1.8	0.6	0.5	5.2	3.2	**1.6**	2.9	2.5	1.4	3.7	4.2	**-1.8**	-1.1	-1.9	-0.9	1.5
Mali	7.4	**2.0**	0.6	0.2	0.6	4.5	5.5	**1.4**	1.6	2.0	0.8	4.0	2.0	**0.6**	-0.9	-1.8	-0.3	0.5
Senegal	8.2	**0.0**	1.0	0.7	0.5	5.0	4.8	**-0.7**	4.0	1.9	0.9	4.3	3.4	**0.7**	-3.0	-1.3	-0.4	0.6
**Group average**	**7.6**	**0.5**	**1.2**	**0.5**	**0.5**	**5.0**	**4.2**	**0.8**	**2.4**	**2.6**	**1.1**	**4.2**	**3.3**	**-0.4**	**-1.2**	**-2.1**	**-0.6**	**0.8**
Kenya	8.2	**-1.6**	2.3	0.9	0.7	4.8	4.0	**0.1**	2.0	3.7	1.4	4.1	4.2	**-1.7**	0.3	-2.8	-0.8	0.7
Malawi	7.9	**-2.2**	1.6	2.0	0.6	5.3	4.7	**-0.8**	1.9	3.8	1.2	4.4	3.2	**-1.4**	-0.3	-1.8	-0.5	0.9
**Group average**	**8.0**	**-1.9**	**1.9**	**1.5**	**0.6**	**5.1**	**4.4**	**-0.3**	**1.9**	**3.8**	**1.3**	**4.3**	**3.7**	**-1.6**	**0.0**	**-2.3**	**-0.6**	**0.8**
Cameroon	7.9	**0.5**	1.2	0.2	0.6	4.9	3.7	**1.8**	1.5	2.9	1.5	3.9	4.1	**-1.2**	-0.2	-2.8	-0.9	1.0
Guinea	6.9	**1.4**	0.7	0.1	0.5	5.8	4.1	**1.4**	1.8	1.3	0.8	6.0	2.8	**0.0**	-1.2	-1.2	-0.3	-0.2
Mozambique	8.1	**0.0**	1.3	0.2	0.6	5.1	3.8	**1.5**	1.2	2.4	1.3	5.1	4.3	**-1.5**	0.1	-2.2	-0.6	0.0
Nigeria	7.8	**1.3**	0.6	0.1	0.5	5.1	4.6	**0.9**	2.1	3.1	1.0	3.5	3.1	**0.4**	-1.5	-3.0	-0.5	1.6
Uganda	8.7	**-1.4**	1.5	0.7	0.6	5.2	5.1	**-0.2**	1.9	3.5	1.2	3.8	3.6	**-1.2**	-0.4	-2.8	-0.6	1.4
Zambia	8.4	**-1.9**	1.2	1.7	0.6	5.2	4.4	**-1.3**	2.6	4.0	1.3	4.2	4.0	**-0.6**	-1.3	-2.3	-0.7	1.0
**Group average**	**7.9**	**0.0**	**1.1**	**0.5**	**0.6**	**5.2**	**4.3**	**0.7**	**1.8**	**2.9**	**1.2**	**4.4**	**3.6**	**-0.7**	**-0.8**	**-2.4**	**-0.6**	**0.8**
Namibia	5.9	**0.0**	1.4	2.7	0.4	4.9	2.7	**0.6**	1.0	5.6	0.8	4.6	3.2	**-0.6**	0.4	-2.9	-0.4	0.2
**Group average**	**5.9**	**0.0**	**1.4**	**2.7**	**0.4**	**4.9**	**2.7**	**0.6**	**1.0**	**5.6**	**0.8**	**4.6**	**3.2**	**-0.6**	**0.4**	**-2.9**	**-0.4**	**0.2**
Madagascar	7.4	**0.4**	1.3	1.1	0.8	4.4	3.3	**1.8**	1.5	3.5	1.9	3.3	4.1	**-1.5**	-0.2	-2.4	-1.1	1.1
Niger	8.7	**0.6**	0.6	0.1	0.5	4.8	6.9	**0.5**	1.9	1.6	0.7	3.8	1.8	**0.2**	-1.3	-1.5	-0.2	1.0
Rwanda	7.5	**-3.1**	3.0	1.7	0.6	5.5	5.5	**-2.7**	3.9	3.5	1.1	4.0	1.9	**-0.4**	-0.9	-1.8	-0.4	1.6
Zimbabwe	5.7	**0.1**	1.4	2.4	0.9	4.8	3.4	**-0.5**	2.8	3.9	1.6	4.1	2.3	**0.6**	-1.4	-1.5	-0.7	0.7
**Group average**	**7.3**	**-0.5**	**1.6**	**1.3**	**0.7**	**4.9**	**4.8**	**-0.2**	**2.5**	**3.1**	**1.3**	**3.8**	**2.5**	**-0.3**	**-1.0**	**-1.8**	**-0.6**	**1.1**
Benin	6.9	**1.0**	1.1	0.3	0.5	5.5	4.7	**1.7**	2.2	1.7	1.0	4.0	2.2	**-0.8**	-1.1	-1.4	-0.5	1.5
**Group average**	**6.9**	**1.0**	**1.1**	**0.3**	**0.5**	**5.5**	**4.7**	**1.7**	**2.2**	**1.7**	**1.0**	**4.0**	**2.2**	**-0.8**	**-1.1**	**-1.4**	**-0.5**	**1.5**
Tanzania	7.6	**-0.2**	1.2	1.1	0.7	4.9	4.6	**-0.1**	1.9	3.7	1.3	4.0	3.0	**0.0**	-0.7	-2.6	-0.6	1.0
**Group average**	**7.6**	**-0.2**	**1.2**	**1.1**	**0.7**	**4.9**	**4.6**	**-0.1**	**1.9**	**3.7**	**1.3**	**4.0**	**3.0**	**0.0**	**-0.7**	**-2.6**	**-0.6**	**1.0**
**Total average**	**7.6**	**-0.1**	**1.3**	**0.9**	**0.6**	**5.0**	**4.3**	**0.5**	**2.1**	**3.0**	**1.2**	**4.2**	**3.2**	**-0.6**	**-0.8**	**-2.1**	**-0.6**	**0.9**

Note: TFR = total fertility rate, R = residual, M = Cm marriage and exposure index, C = Cc contraceptive use index, A = Ca abortion index, I = Ci postpartum infecundability index

#### Earliest surveys

We take the example of Mozambique in Table 3. In the earliest survey, women in the poorest wealth quintile had a total fertility rate of 6.6. For these women, postpartum infecundability (postpartum amenorrhea and postpartum abstinence) brought the total fertility rate down by 4.5 from the biological maximum of 15.3. Abortion brought the total fertility rate down by a further 0.9. Contraception brought the total fertility rate down by 0.1, and non-exposure (for example, delayed marriage) brought fertility down a further 1.0. The unexplained residual brought fertility down by 2.3.

That is, for the poorest in Mozambique in the earliest survey, postpartum infecundability and delayed marriage were the most effective methods in bringing fertility rates down from the biological maximum. Contraceptive use played a minor role.

For the richest wealth quintile in the earliest survey in Mozambique, the observed total fertility rate was 4.9. For these women, postpartum abstinence and amenorrhea reduced the total fertility rate by 4.6 from the biological maximum of 15.3. Abortion reduced the total fertility rate by 1.2. Contraceptive use reduced the total fertility rate by 1.5. Non-exposure (for example, delayed marriage) brought fertility down by a further 1.2. The unexplained residual brought fertility down by 1.9.

In the earliest survey in Mozambique, the rich (4.6) relied on postpartum infecundability more than the poor (4.5) in reducing the total fertility rate from the biological maximum. The rich (1.5) used contraception more than the poor (0.1) to reduce fertility from the biological maximum. The rich (1.2) also saw non-exposure to sex as a determinant of their fertility rates more than the poor (1.0). The unexplained residual brought fertility down from the biological maximum for both the rich and poor, but it was larger for the poor (2.3) than the rich (1.9).

#### Latest survey

In the latest survey for Mozambique, Table 4, the observed total fertility rate went up for the poorest (6.6 to 8.1) and went down for the richest (4.9 to 3.8)–the gap in the total fertility rate between rich and poor widens due to an increase in fertility rate of the poorest, combined with a decrease in the fertility rate of the richest over time.

For Mozambique, not only did the total fertility rate of the richest and poorest change over time, but so too did the proximate determinants of fertility.

Postpartum infecundability became an increasingly important factor to bring down the total fertility rate from the biological maximum for both the rich (4.6 to 5.1) and the poor (4.5 to 5.1). The influence of contraceptive use in reducing fertility from the biological maximum increased for the rich from 1.5 to 2.4 and for the poor from 0.1 to 0.2. The influence of delayed marriage did not change for the richest, remaining at 1.2 over time, but increased for the poorest (1.0 to 1.3).

To summarize, postpartum infecundability became important to both the rich and the poor in Mozambique. The rich used contraception much more than the poor. There was little change over time in the forces of delayed marriage for the rich, but slightly positive for the poor.

### Inequality in the proximate determinants of fertility increasing or decreasing over time, the case of Mozambique

In Table 5, the *gap* between rich and poor total fertility rate (Rodriguez) increased for Mozambique between the earliest and latest surveys. The poor relied on delayed marriage more than the rich did. However, the rich utilized contraception more than the poor did.

**Table 5:** Proximate determinants of fertility by wealth quintile, over time.

	Latest Poorest–Earliest Poorest						Latest Richest–Earliest Richest						Latest (Poorest–Richest)–Earliest (Poorest–Richest)					
	TFR	R	M	C	A	I	TFR	R	M	C	A	I	TFR	R	M	C	A	I
Burkina Faso	-0.3	**0.4**	0.6	0.3	0.0	-1.1	-1.2	**0.4**	0.6	1.6	0.2	-1.6	0.9	**0.0**	0.1	-1.3	-0.2	0.5
Cote d'Ivoire	-0.2	**0.1**	0.2	0.3	-0.1	-0.4	-1.0	**0.5**	0.5	0.2	0.0	-0.3	0.8	**-0.4**	-0.4	0.1	-0.1	0.0
Ethiopia	-0.1	**0.9**	-0.6	0.6	0.1	-0.8	-0.6	**-0.3**	-0.4	2.2	0.4	-1.3	0.5	**1.1**	-0.2	-1.6	-0.3	0.5
Ghana	-0.2	**-0.5**	0.6	0.1	-0.2	0.2	-0.9	**-0.4**	1.3	0.5	0.1	-0.5	0.7	**-0.1**	-0.7	-0.4	-0.2	0.6
Mali	-0.4	**0.2**	0.1	0.1	-0.2	0.1	-0.7	**0.3**	0.4	0.2	-0.1	0.0	0.3	**-0.1**	-0.3	-0.1	0.0	0.2
Senegal	-0.1	**0.0**	0.1	0.6	-0.1	-0.6	-0.7	**0.1**	0.4	0.3	-0.1	-0.1	0.6	**-0.1**	-0.3	0.3	0.0	-0.5
**Group Average**	**-0.2**	**0.2**	**0.2**	**0.3**	**-0.1**	**-0.4**	**-0.8**	**0.1**	**0.5**	**0.8**	**0.1**	**-0.6**	**0.6**	**0.1**	**-0.3**	**-0.5**	**-0.1**	**0.2**
Kenya	0.02	**-1.0**	0.9	0.2	0.0	-0.1	-0.4	**-0.1**	0.2	0.2	0.0	0.1	0.4	**-0.9**	0.7	0.0	0.0	-0.2
Malawi	0.03	**-1.8**	-0.6	1.5	-0.2	1.0	-1.7	**-0.8**	0.1	2.0	0.2	0.3	1.8	**-1.0**	-0.7	-0.5	-0.3	0.7
**Group average**	**0.0**	**-1.4**	**0.2**	**0.9**	**-0.1**	**0.5**	**-1.1**	**-0.5**	**0.2**	**1.1**	**0.1**	**0.2**	**1.1**	**-0.9**	**0.0**	**-0.2**	**-0.2**	**0.3**
Cameroon	1.43	**-1.5**	0.6	0.0	0.0	-0.6	-1.3	**-0.2**	0.8	0.9	0.5	-0.7	2.8	**-1.2**	-0.2	-0.9	-0.5	0.1
Guinea	0.45	**0.0**	0.2	0.0	0.1	-0.8	-0.6	**0.0**	0.6	0.3	0.2	-0.4	1.1	**0.1**	-0.4	-0.2	-0.1	-0.4
Mozambique	1.5	**-2.2**	0.3	0.1	-0.3	0.6	-1.0	**-0.4**	0.0	0.9	0.1	0.5	2.5	**-1.9**	0.3	-0.8	-0.4	0.2
Nigeria	0.73	**0.1**	-0.1	0.0	-0.1	-0.6	-1.2	**0.0**	1.0	1.8	0.2	-1.8	1.9	**0.1**	-1.1	-1.8	-0.4	1.2
Uganda	1.09	**-2.6**	0.8	0.5	-0.2	0.4	-0.8	**-0.9**	0.6	1.2	0.1	-0.3	1.9	**-1.7**	0.2	-0.8	-0.3	0.7
Zambia	0.71	**-1.9**	0.1	1.2	-0.1	0.0	-1.3	**-0.3**	0.2	1.4	0.3	-0.2	2.0	**-1.6**	-0.1	-0.1	-0.4	0.1
**Group average**	**1.0**	**-1.3**	**0.3**	**0.3**	**-0.1**	**-0.2**	**-1.0**	**-0.3**	**0.5**	**1.1**	**0.2**	**-0.5**	**2.0**	**-1.0**	**-0.2**	**-0.8**	**-0.3**	**0.3**
Namibia	-1.3	**-0.6**	-0.6	2.3	0.0	0.2	-1.3	**0.2**	-0.2	0.9	0.1	0.2	0.0	**-0.8**	-0.4	1.3	-0.1	0.0
**Group average**	**-1.3**	**-0.6**	**-0.6**	**2.3**	**0.0**	**0.2**	**-1.3**	**0.2**	**-0.2**	**0.9**	**0.1**	**0.2**	**0.0**	**-0.8**	**-0.4**	**1.3**	**-0.1**	**0.0**
Madagascar	-1.7	**0.7**	0.1	0.9	0.1	-0.2	-0.8	**0.6**	-0.7	0.8	0.3	-0.2	-0.9	**0.1**	0.8	0.1	-0.1	0.0
Niger	-0.4	**0.8**	-0.2	0.1	-0.1	-0.1	-0.1	**0.8**	-0.2	0.1	-0.1	-0.5	-0.3	**0.0**	0.0	0.0	0.0	0.3
Rwanda	-1.9	**0.1**	0.3	1.0	0.0	0.5	-1.3	**-0.4**	0.2	1.9	0.2	-0.7	-0.7	**0.5**	0.1	-1.0	-0.2	1.2
Zimbabwe	-1.4	**0.4**	-0.2	0.7	0.1	0.5	-0.6	**0.1**	0.6	-0.2	0.1	0.0	-0.9	**0.3**	-0.8	0.9	0.0	0.5
**Group average**	**-1.4**	**0.5**	**0.0**	**0.7**	**0.0**	**0.2**	**-0.7**	**0.3**	**0.0**	**0.7**	**0.1**	**-0.3**	**-0.7**	**0.2**	**0.0**	**0.0**	**-0.1**	**0.5**
Benin	-1.1	**0.3**	0.5	0.1	-0.1	0.3	0.0	**-0.2**	0.8	0.0	-0.2	-0.4	-1.1	**0.5**	-0.3	0.0	0.1	0.7
**Group average**	**-1.1**	**0.3**	**0.5**	**0.1**	**-0.1**	**0.3**	**0.0**	**-0.2**	**0.8**	**0.0**	**-0.2**	**-0.4**	**-1.1**	**0.5**	**-0.3**	**0.0**	**0.1**	**0.7**
Tanzania	-0.5	**0.1**	0.3	0.7	0.1	-0.6	0.1	**-1.4**	0.9	1.5	0.1	-1.2	-0.6	**1.4**	-0.6	-0.7	-0.1	0.6
**Group average**	**-0.5**	**0.1**	**0.3**	**0.7**	**0.1**	**-0.6**	**0.1**	**-1.4**	**0.9**	**1.5**	**0.1**	**-1.2**	**-0.6**	**1.4**	**-0.6**	**-0.7**	**-0.1**	**0.6**
**Total average**	**-0.2**	**-0.4**	**0.2**	**0.5**	**-0.1**	**-0.1**	**-0.8**	**-0.1**	**0.4**	**0.9**	**0.1**	**-0.4**	**0.7**	**-0.3**	**-0.2**	**-0.3**	**-0.2**	**0.3**

Note: TFR = total fertility rate, R = residual, M = Cm marriage and exposure index, C = Cc contraceptive use index, A = Ca abortion index, I = Ci postpartum infecundability index

In Table 5, we see the summary for each country *changes in the gap* in total fertility rate and *changes in gap* in the proximate determinants of fertility. In the first column, for the total fertility rate (Rodriguez), a negative value indicates that the gap between richest and poorest total fertility rate is closing–declining inequality in the total fertility rate between richest and poorest. A positive value indicates that the inequality in fertility between richest and poorest is increasing. Mozambique saw an increase in the gap between richest and poorest total fertility rate over time of 2.5 children per woman–increasing inequality.

For the residual R, a negative value indicates that the proximate determinant has a bigger impact on observed fertility rates for the richest compared to the poorest. A positive value indicates that the proximate determinant has a bigger impact over time on observed fertility rates for the poorest compared to the richest.

Of the 21 countries, 14 countries saw the richest rely on delayed marriage at an increasing rate compared to the poorest in reducing fertility from the biological maximum.

Of the 21 countries, 13 countries saw the rich use contraception at an increasing rate compared to the poor in reducing fertility from the biological maximum.

Of the 21 countries, 16 countries saw the poor rely on postpartum infecundability at an increasing rate relative to the rich in reducing fertility from the biological maximum. (The poor relied on postpartum infecundability at an increasing rate relative to the rich, and yet the poor still fell behind in terms of total fertility rate).

Of the 21 countries, 11 countries saw the rich unexplained residual reducing fertility at a greater rate than the poorest.

For 14/21 countries, the gap between richest and poorest total fertility increased (Burkina Faso, Cameroon, Cote d’Ivoire, Ethiopia, Ghana, Guinea, Kenya, Malawi, Mali, Mozambique, Nigeria, Senegal, Uganda, and Zambia). For these countries, the richest saw increases in contraceptive use and age of exposure (delayed marriage) to a greater extent than the poorest. This puts more downward pressure on the total fertility rate of the richest than the poorest through these two proximate determinants. Moreover, for this group on average, both the poorest and richest decreased their reliance on postpartum infecundability (putting upward pressure on the total fertility rate). However, this decreased reliance on postpartum infecundability did not offset the role of delayed marriage and contraction in decreasing the total fertility rate. The poor also reduced their reliance on postpartum infecundability by less than the richest.

For the group of countries that saw an increase in fertility inequality due to the richest decreasing in the total fertility rate while the poorest increasing their total fertility rate (the only group that actually saw the poorest increase in their total fertility rate over time), delayed marriage and contraceptive use both increased in their role for the richest and poorest putting downward pressure on total fertility rate for both wealth groups. The upward pressure on total fertility rate created by increased use of postpartum infecundability for the richest and poorest, did not sufficiently offset the role of contraception and delayed marriage to cause the increase in total fertility rate for the poorest. In this case, it was the role of the residual, which saw total fertility rate of the poorest increase, highlighting the need to discover what this residual represents.

For Kenya and Malawi, these counties also saw an increase in the inequality between richest and poorest for the total fertility rates–driven by the richest decreasing in total fertility rate while the poorest remained unchanged (rather, it was a very small increase) in their total fertility rate over time. For these two countries, the richest increased in their reliance on delayed marriage. The poorest in Malawi saw a decrease in age of marriage or sexual debut between the latest and earliest surveys. The richest and poorest in both countries saw an increased role of contraceptive use; this was stronger for the richest in Malawi. In Kenya, the poorest decreasingly relied on postpartum infecundability, while the poorest and richest in Malawi increased in their reliance on postpartum infecundability.

For Namibia, where the richest and poorest declined equally in their total fertility rate, age of marriage/exposure actually declined for both groups (poorest more than richest) putting upward pressure on total fertility rate. However, in Namibia, contraceptive use of the poorest increased as a rate greater than the increase for the richest. This catch-up in contraceptive use of the poorest meant that both the richest and poorest saw equal decline in their total fertility rates.

For six countries, the gap between richest and poorest total fertility rate decreased. For Madagascar, Niger, Rwanda, and Zimbabwe, *on average* there was no change in age of marriage, equal increase in contraceptive use by richest and poorest, and a stronger role of postpartum infecundability for the poorest than the richest. Postpartum infecundability played the role of closing the gap between richest and poorest total fertility rate.

For Benin, the richest saw no decline in their total fertility rate while the poorest saw a decline in total fertility rate. The gap between rich and poor total fertility rate narrowed. For the poorest there was increased reliance on delayed marriage, contraceptive use, and postpartum infecundability (and the residual), and thus all the proximate determinants worked to decrease total fertility rate of the poorest. However, the richest saw no change in their contraceptive use, a declining dependence on postpartum infecundability (upward pressure on total fertility rate). Age of marriage/exposure of the richest increased (downward pressure on total fertility rate).

For Tanzania, where the poorest saw a decrease in their total fertility rate and the richest an increase in their total fertility rate (and a narrowing of the gap between richest and poorest total fertility rate), this was driven by the unknown residual of the richest.

In summary, we could say that for those countries that saw an increase in inequality of fertility rates, the poorest were declining in their use of postpartum infecundability and did not compensate or keep up with the richest in terms of contraceptive use. Trends in increasing the age of exposure/marriage also lagged for the poorest.

For those countries that saw a decrease in the gap between richest and poorest in terms of the total fertility rate, there was an increased role of postpartum infecundability in reducing fertility rates for the poorest. Moreover, contraceptive use of the poorest for these countries increased and kept pace with the richest. Delayed marriage played a minor (or non-existent) role.

## Discussion

To investigate why fertility inequality changed differently for the richest and poorest countries, we used the Bongaarts’ proximate determinants of fertility model, in which these proximate determinants of fertility directly determine the fertility rate. In this paper, we found that there was increasing inequality in the fertility rate across wealth quintiles for 14/21 SSA countries. We assume that the proximate determinants of the levels of fertility are also the proximate determinants of inequality in fertility. Such that, inequality in sexual exposure, inequality in contraceptive use, and inequality in postpartum infecundability lead to inequality in fertility rates.

We found that increasing inequality in the total fertility rate was driven by positive trends in delayed marriage for the richest wealth quintile and modest gains in contraceptive use by the richest. However, the poorest did not see these gains, hence the rising inequality the total fertility rate between the richest and poorest. Rossier et al [24] have noted the smaller role of modern contraceptive use in determining observed fertility of the poorest. Applying Bongaarts’ model, and putting exposure, contraceptive use, abortion, postpartum abstinence, and amenorrhea (breastfeeding) side-by-side, our descriptive data analysis also highlights the modest role of modern contraceptive use in determining observed fertility of the poorest. Moreover, there is a significant role of delayed marriage for the richest, and postpartum infecundability for the poorest in determining fertility rates.

Postpartum infecundability has played a very large role in reducing fertility rates for the poorest wealth quintile (although gains were not enough to see significant declining inequality in fertility). Without this important mechanism of postpartum infecundability, there would have been a much greater divergence of the fertility rate between the richest and poorest. As Rossier and Corker [24] pointed out, natural methods of fertility regulation play an important role in sub-Saharan Africa, and in this paper we show that postpartum abstinence and breastfeeding are vital methods for the poorest, in particular, in regulating fertility.

Research typically focuses on the role of early marriage (sexual exposure), contraceptive access and use, and programs designed to foster these fertility regulating instruments [25]. However, in our application of Bongaarts’ model, we did not ignore the role of postpartum infecundability and, indeed, we show in this paper, that it is crucial in observing some fertility rate decline for the poor.

Moreover, through the descriptive analysis in this study, we have found that the residual proximate determinant—which is yet unknown—has played an increasingly important role in how the rich move away from the poor in terms of fertility decline. Further work is needed to fully understand what the residual is.

Following from the results of this study, the implications for policy are: 1) programs targeting delayed marriage need to be inclusive of the poorest wealth quintile to ensure that fertility decline through this mechanism is experienced across the wealth spectrum; 2) programs promoting contraceptive use for the poorest will help with an equal decline in the total fertility rate; and, 3) postpartum infecundability is employed very effectively by the poor, and when it is promoted for the poor, it helps close the gap in total fertility rate. In fact, when postpartum infecundability is de-emphasized, inequality in total fertility rate increases.

While some previous research does emphasize the role of postpartum infecundability in fertility regulation in poor resource settings [26, 27], policies that deemphasize postpartum infecundability (including breastfeeding and lactation amenorrhea) in turn deemphasize important fertility regulation methods that the poorest are already trying to use. Efforts should be made to capitalize on these existing trends by the poorest quintile to foster breastfeeding programs that already work well. This, of course, overlaps with the larger driving agenda to couple maternal health (and child health) services with reproductive services at the clinic level.

In addition to the higher-level limitations addressed in the introduction, there are limitations to this analysis that should be recognized so that interpreting the results can be made with caution. The residual was often high, and we do not fully understand its composition. Misreporting and potentially under-reporting of contraceptive use would downplay the contribution of contraception to the determination of the observed total fertility rate. In addition, the abortion rate we used was for the entire region and was not country and age specific, as the other components were calculated. This means that our interpretation of abortion rates was not specific to countries or wealth quintiles.

Despite these limitations, this paper has shown that inequality in fertility is increasing in 14 out of the 21 sample countries. This divergence in fertility rates between the richest and poorest wealth quintiles is due to the richest wealth quintile’s fertility decline outpacing the fertility decline of the poorest in 6/14 countries, and the poorest increasing in their total fertility rate compared to declines in the richest quintile in 6/14 countries. The fertility decline of the richest is driven by delayed marriage and modest increases in contraceptive use. Likewise, for the richest, postpartum abstinence and postpartum amenorrhea played a decreasing role. However, for the poorest, postpartum abstinence and postpartum amenorrhea played an increasingly important role in reducing fertility rates (although not sufficient to reduce inequality in total fertility rates).

Without Bongaarts’ proximate determinants framework, the policy focus is on child marriage and contraceptive access in completely different fields. This paper, however, shows that natural methods of fertility regulation are important in reducing fertility rates–especially for the poor. Any programmatic or policy shift away from these natural methods will see an increase in inequality in fertility rates.
